# FORESAM—FOG Paradigm-Based Resource Allocation Mechanism for Vehicular Clouds

**DOI:** 10.3390/s21155028

**Published:** 2021-07-24

**Authors:** Rickson Pereira, Azzedine Boukerche, Marco A. C. da Silva, Luis H. V. Nakamura, Heitor Freitas, Geraldo P. Rocha Filho, Rodolfo I. Meneguette

**Affiliations:** 1 Department of Computer Science, São Paulo State University—UNESP, São José do Rio Preto 15054-000, Brazil; rickson.simioni@unesp.br; 2 School of Electrical Engineering and Computer Science, University of Ottawa, Ottawa, ON K1N 6N5, Canada; aboukerc@uOttawa.ca; 3 Institute of Mathematical and Computer Sciences, University of São Paulo, São Carlos 13566-590, Brazil; marco.colombo@usp.br; 4 Federal Institute of São Paulo (IFSP), Araraquara 15808-305, Brazil; nakamura@ifsp.edu.br; 5 Sidia, Institute of Science and Technology, Manaus 69055-035, Brazil; heitor.vieira@sidia.com; 6 Department of Computer Science, University of Brasília, Distrito Federal, Brazilia 70910-900, Brazil; geraldof@unb.br

**Keywords:** intelligent transportation systems, mobile cloud, resource allocation, vehicular cloud

## Abstract

The Intelligent Transport Systems (ITS) has the objective quality of transportation improvement through transportation system monitoring and management and makes the trip more comfortable and safer for drivers and passengers. The mobile clouds can assist the ITS in handling the resource management problem. However, resource allocation management in an ITS is challenging due to vehicular network characteristics, such as high mobility and dynamic topology. With that in mind, we propose the FORESAM, a mechanism for resources management and allocation based on a set of FOGs which control vehicular cloud resources in the urban environment. The mechanism is based on a more accurate mathematical model (Multiple Attribute Decision), which aims to assist the allocation decision of resources set that meets the period requested service. The simulation results have shown that the proposed solution allows a higher number of services, reducing the number of locks of services with its accuracy. Furthermore, its resource allocation is more balanced the provided a smaller amount of discarded services.

## 1. Introduction

The current tendency is to provide vehicles and roads with resources to make the transport infrastructure safe, efficient, and helpful for passengers. Respectively, the first implies giving information about traffic jams, accidents, dangerous conditions in the road, weather conditions, and restaurants and gas stations nearby [1,2]. The second means to increase the road network’s capacity, reduce air pollution and traffic congestion, reduce operating costs of vehicles, and make more efficient the public transport system and road logistics [3,4,5]. Finally, the last implies providing internet access for several applications, such as tourism/advertising information, chat, and download files. These applications are typical examples of Intelligent Transport Systems (ITS). Its goals are to enhance the transport system’s efficiency, security, and entertainment through new information and communication technologies [6,7].

A definition for ITS can be defined as an aggregation of communication technologies and computational resources to optimize a city’s transportation system [8,9]. To do this, ITS intercommunication between vehicles with a shoulder infrastructure that can be a mobile phone network or a wireless network to capture vehicle information and traffic [10,11]. However, with the increase of vehicles on urban roads, there is also an increase in the flow of information generated by these vehicles that send and receive not only traffic information but also information from other categories of services, such as entertainment and yellow page services [12,13].

Linking vehicle growth to increased information flow, a study by the United States Transit Department describes an increase of 10 million vehicles from 2010 to 2014 [14]. An IHF Automotive study [15] estimated that in-vehicle devices transferred over 480 data terabytes through over 26 million connections. That same study predicted that about 30 data terabytes would be transmitted a day through about 152 million connections by 2020.

These numbers can be higher once we have autonomous cars that are vehicles that have a maintenance system that changes lanes; a speed controller (with automatic brake, deceleration, and acceleration promoter), sensors, and radars that detect the surroundings; a high-resolution GPS (capable of reading the exact lane the vehicle is in and the correct exit location on a highway, for example); powerful cameras; and, of course, an advanced artificial intelligence system that can make decisions on the spot of the human driver. Therefore, the development of new infrastructure, traffic management mechanisms, and computational resources are necessary to meet ITS services [11].

One possible solution is sharing computational capacities, such as storage and processing, with nearby vehicles [16]. One possibility is the use of vehicular cloud, which consists of a set of cooperating vehicles and collaboration among themselves sharing their computational resources, which can be dynamically scheduled between vehicles or by roadside units (RSU) [17]. To assist the vehicular cloud in managing available resources and offer a more extensive range of services without impacting the network and user experience, the FOG paradigm is used [18]. Furthermore, FOG can aggregate more computational resources, assisting in the processing of requests, and it also assists in the allocation and the acquisition of resources to better fit the cloud service request [19,20].

In the global research area, the allocation of resources in the vehicular cloud is already explored through some works [21,22,23,24,25,26,27,28,29,30,31,32]. Pereira et al. proposed a resource allocation policy for vehicular clouds in the highway environment. However, this approach did not consider aspects of communication in an urban environment. Dai et al. proposed mathematical models to solve the problems with allocating in-vehicle networks that used the direct communication between the RSU and the vehicle. Meng et al. [26] solved the resource allocation problem using the method of the Semi-Markov Decision Process (SMDP). Yu et al. [29] proposed the use of the game theory to carry out vehicular cloud resource management. Arkian et al. [27] proposed the use of fuzzy logic to address the problem of resource allocation. These works do not use all elements of a cloud carrier’s infrastructure to carry out the allocation and aggregation of resources in the cloud.

To overcome these challenges, we propose a system for managing the resources in the vehicle cloud context, called FORESAM—FOG paradigm-based Resource Allocation Mechanism for vehicular clouds. FORESAM considered FOGs to aim in the management of available resources. Considering that vehicles have embedded resources, such as storage and processing, added to the FOG-managed vehicle cloud resource pool. Therefore, vehicles must collaborate with the creation of a resources pool to realize available resources, which will be serviced by vehicles and the FOG set that will manage the resources and the attempt of the services. Thus, FORESAM will increase the usability of the features in the vehicular cloud.

FOGs allow the aggregation of its idle resources with vehicle resources, increasing the service availability capacity. In addition, the FOG’s approach to vehicles makes FOG an essential element for the cloud, providing a low communication latency.

FORESAM increases the usability of the computer capacity provided by the vehicle, besides managing the requested services. To this end, FORESAM uses a mathematical model and a multi-criteria mathematical method to make the best choice of allocation while considering the parameters required for service. This model also aims to perform resource availability allocation control.

For the FORESAM assessment, we compared it with other resource allocation methods. FORESAM provided an increase in the number of services offered and reduced the locks and the number of not attempted services. This occurs because of the load balancing in the allocation of resources that FORESAM provides.

The main contributions of this paper are as follows:

(i) The development of a FOG-based mechanism for the allocation and aggregation of vehicular cloud resources;
(ii) The development and evaluation of a decision-making policy for the allocation of resources based on the resources required for the proper execution of the requested service; and
(iii) FORESAM validation through real mobility trades, aiming to bring more realistic results.

This paper’s remainder is structured as follows. We describe the most relevant related solutions in Section 2. We present our proposed protocol for resource allocation in the vehicular cloud in Section 3. We describe the validation of the propulsion mechanism and its results in Section 4. Finally, we summarize our work and provide direction for future works in Section 5.

## 2. Related Works

Several papers in the literature propose a solution to the problem of vehicular cloud resource allocation [21,23,24,25,26,33,34,35].

Da Costa et al. [36] proposed a vehicle cloud allocation mechanism called CRATOS (CombinatoRial optimization-bAsed Task allOcation mechaniSm for VC). CRATOS uses the 0/1 Knapsack theory to make the decision to allocate resources to meet the requested service. CRATOS is centralized in which there is a controller element called controller located at a higher level in the network in which it processes requests, in which it assumes that the services to be allocated have a defined weight. Although you can control the allocated services, this technique does not optimize the use of available resources in the vehicular cloud.

Hattab et al. [37] proposed a mechanism for allocating computational resources in the vehicular cloud based on the polynomial-time complexity algorithm. This approach uses the queuing theory to perform the allocation, which is performed by cooperation between vehicles. The authors did not consider the mobility of vehicles for the VC formation; VCs are stationary.

Tang et al. [38] proposed a centralized allocation mechanism called DbHA (Distance Based Heuristic Algorithm for Utility Optimization). DbHA is a greedy strategy algorithm that considers a utility function to perform the resource allocation decision in VC. DbHA considers the parameters, such as the computing and communication resources, required by the tasks for the decision-making process. The authors calculate the Hessian matrix of a function with such variables and obtain the optimal values of computation and communication when the maximum value of the utility function is reached.

Garg et al. [39] proposed a resource allocation method called HLMS. HLMS is an improvement in the work of Ashok et al. [40]. In this method, the map is also divided into four areas. Vehicles in the center of each area are ranked as level one leaders. The servers are selected from the intersection density that the area has and Ashok’s work. In addition to the server selection process, the HLMS also succumbs to the Ashok service search and allocation procedure. The vehicle will send the first phase leader first, and if the request cannot be found, then the vehicle will send it to the second level server. Although it finds an improvement in the search method and service allocation, HLMS still has the same problems as Ashok’s work.

Tao et al. [24] have put in place a Gauss–Seidel (GS) based on a cloud resource allocation mechanism. These mechanisms are intended to reduce the Nash Equilibrium Point (NEP) calculation by considering each service process’s inbound and outbound scheduling and modulating a flow interaction control mechanism. Therefore, this mechanism uses the game theory to allocate the resource in the vehicular cloud by modeling the vehicle’s packet transmission. This mechanism also uses the RSU to facilitate communication between the data center and vehicle to assist in the allocation process. Thus, the vehicles try to minimize their cost.

Yu et al. [21] have provided a resource allocation mechanism in the vehicular cloud. This approach uses a central cloud to decide whether or not to allocate the resource. This decision is made through a game-theoretical mechanism to address the resource problem. For the vehicle to have access to services available through the cloud, the vehicle must be connected to the data center through an RSU. As the distance between the data center and the vehicle increases, the communication between the two can increasingly delay, or the latency can increase when servicing a particular service.

Meneguette et al. [23] proposed a mechanism for allocating and managing services based on the communication between vehicles only, using the semi-Markov decision process as the decision mechanism of whether or not the vehicle should allocate the resource. To this end, a cluster is established, in which the cluster head checks whether or not to serve the service. Therefore, this approach does not consider a set of RSUs to assist in vehicle resource management and communication, which can create higher overhead for creating and maintaining this vehicle-to-vehicle communication infrastructure. Moreover, this solution does not consider some critical parameters, such as service time and processing time. Deciding whether to transfer services between clouds could be more difficult because of this.

Some of the work described considers using a central cloud, in which they perform all of the management, which may increase the latency of the service provided due to the distance between the vehicles and the cloud. Other work uses only the cooperation between vehicles to create and manage resources. However, such a solution may generate higher overhead to keep the denial allocation infrastructure active. Table 1 shows a comparison of the literature work described with the proposed solution. Thus, in this paper, we use the FOG paradigm to decrease the latency of allocated services and decrease the overhead due to the support infrastructure in the aggregation and communication of resources made available by vehicles.

## 3. FORESAM—FOG Paradigm-Based Resource Allocation Mechanism for Vehicular Clouds

**Figure 1:**
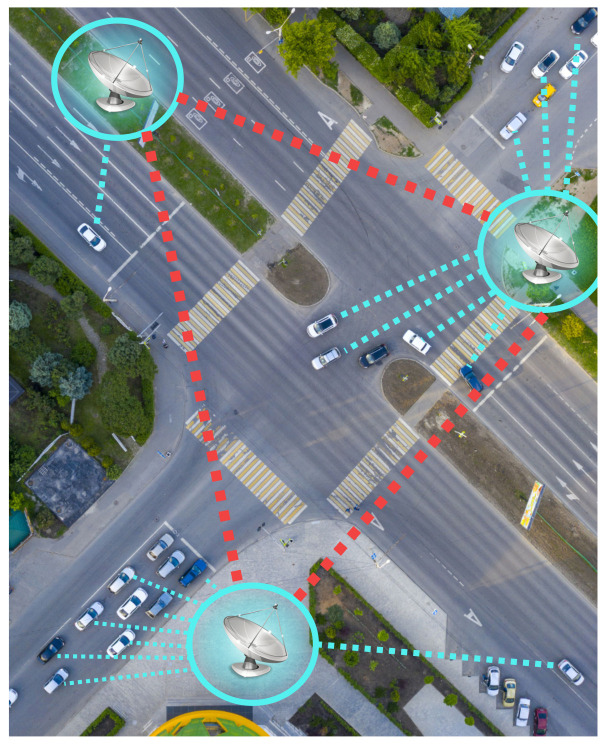
The vehicular cloud environment.

This section describes FORESAM—FOG paradigm-based Resource Allocation Mechanism for vehicular clouds. FORESAM is designed to handle the management and allocation of vehicle resources to manage the use of vehicle resources quickly due to the dynamic topology of a vehicle network. A hierarchical analytical method is used because it has a lower inference rate since the decision matrix is already built, and it should only be applied based on its weights. Given this, we consider vehicles with idle computing resources, such as processing and storage, i.e., storage space and processing time. These resources can be made available for the formation of a vehicular cloud. Figure 1 describes the vehicular cloud environment used in this paper. Therefore, vehicles and FOG’s will form an infrastructure to provide services for other vehicles. Thus, FORESAM will create a vehicular cloud in which resources are shared by vehicles and FOGs configured to offer cloud service to other vehicles.

Therefore, we consider that the vehicular cloud will be composed of a set of FOGs and vehicles in which computational resources will be available. These idle vehicle resources will be added to the FOGs resources, where the FOGs will manage them. We can consider that the FOG would be an adapted RSU with a set of computational resources available in this work. Thus, we also consider that the FOG has computational resources added to the pool of resources. Once these resources are available, the vehicles can allocate them to perform the desired services, such as entertainment services and traffic exchange services, among others.

**Figure 2:**
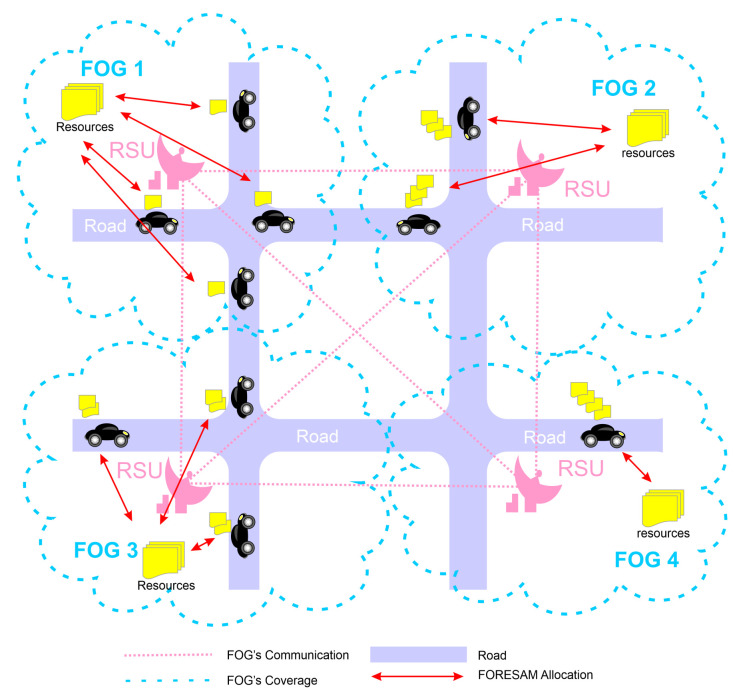
FORESAM abstraction.

Figure 2 describes the FORESAM abstraction in which vehicles are connected to the FOG mirrored on the city map. The intercommunication between vehicles and FOG represented in the image by the antennas and the RSUs provides the infrastructure for aggregating and allocating resources, forming a layer. Therefore, FORESAM provides a layer through the communication between the FOG and the vehicles. The FOG would perform the function of the super-peer, in which it performs all the management in the aggregation of resources in one vehicle network. Vehicles, in turn, provide their available resources for FOGs to allocate to other requesting vehicles.

This set of FOGs can help manage resources by interconnecting with other FOGs, further increasing its knowledge of the resources available to vehicles through a wired connection. However, wireless communication between the set of FOG and vehicles uses the standard for vehicular networks 802.11p. In addition, this work’s focus is not the communication in a vehicular cloud but the management of resources inserted in a vehicular cloud. We will assume that the communication is already being carried out efficiently, and we will prioritize the mechanism only in the allocation and management of such resources.

Each service has a range of computation resources, for example, storage, processing, and run-time, to run efficiently. Thus, the decision on which FOG will allocate resources and whether or not to allocate resources is taken by the FOG.

FOG allocates the idle computational capacity existing in the vehicles and its idle computational resources to provide the resources that are filled by the required services.

To solve the allocation problem, we assumed that *V* vehicles might be communicating with the FOGs. Therefore, these vehicles are sharing their available resources $r_{vehs}$ with the FOGs. Furthermore, to attempt the services requested *s*, the mentioned resources need to be allocated. Therefore, the requested services use $r_{serv}$ resources.

Thus, the vehicle $v_{i}$ traveling in the route requests a service to FOG that, in your turn, allocates computational resources to provide the requested service. In order to meet requests more efficiently, the vehicle could migrate from one FOG to another if the resource is not allocated when it is going through that FOG.

The following sections will describe the communication protocol between FOG and vehicles and the allocation decision mechanism.

### 3.1. Communication Protocol

To communicate between all the elements consisting of the vehicular cloud is necessary to develop an intercommunication protocol between the FOGs and between the FOG and the vehicle, allowing the vehicle to disseminate which computational resources are available for FOG.

The communication protocol is based on the SERVitES protocol [16], which creates a communication infrastructure only between vehicles, not considering the RSUs connection. Thus, we consider the FOG as the cluster head, reducing the number of messages transmitted on the network since all requests initially go to the FOG. In addition to adding communication with the RSU, a minor change to the map split policy was made.

**Figure 3:**
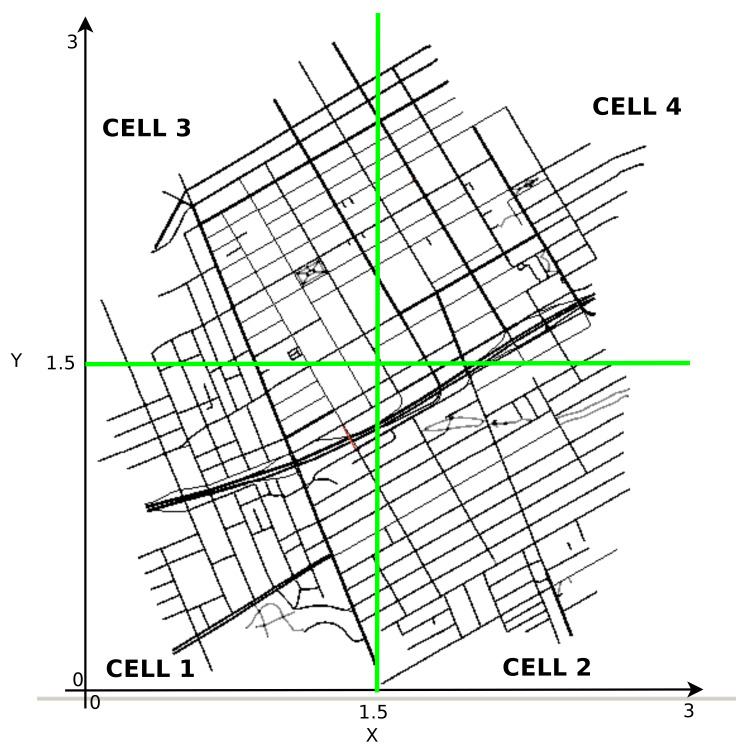
Cells in the map in SERVitES [16].

In SERVitES, the city was previously divided into a set of cells. These cells were divided equally according to the size of the urban area, and the cluster head would be the longest-running vehicle within the cell, as we can see in Figure 3. However, in FORESAM, this cell phone division is by the coverage area of the FOGs, i.e., by the communication radius that the RSU has. Thus, we assume that a set of RSU covers the urban area.

For instance, consider a map 16 km${}^{2}$, 4 km wide, and 4 km long. Assuming that the communication coverage area is RSU is 1Km, we will have 16 RSU and, consequently, 16 FOGs, which each FOG has on its communication area in a set of ways. This knowledge of the roads will allow us to choose a better FOG. In order to make this choice, the vehicle sends a control message from time to time to the beacon containing the vehicle id, the number of available resources, and its trajectory (line 1 of Algorithm 1). FOG receives the message and calculates the time this vehicle will remain and its area based on the vehicle’s trajectory and the streets covered by the FOG communication area (lines 2–3). Thus, FOG knows how long the resource provided by the vehicle will manage. If the time to remain in the FOG is shorter than time to pass through a street segment, the FOG will check the next FOG considering the vehicle’s trajectory and will forward the vehicle control message to approach FOG (lines 4–6). Therefore, speeding up the allocation of resources in a FOG that the vehicle will stay for longer, in addition to reducing the amount of control messages and the FOG change time, since the vehicle’s time would be connected in the first FOG would be minimal. This procedure also occurs when the vehicle is in the overlap between two or more FOG, in which it will chose the FOG that the vehicle will spend by the connected time.
**Algorithm 1: **Communication FOG and Vehicles1: Vehicle_send(Beacon)2: FOG_received(Beacon)3: myFOG=ComputeTime(Veh_Traj)4: **if** (myfoglessthanstreetSegmentTime) **then**5: otherFog=FindNexT(Veh_Traj)6: $FOG\_sendtoz_{o}therFog\left(Beacon\right)$7: **end if**8: ManagerResource

Communication between the FOGs takes place through a wired connection. Thus, when initialized, the FOGs sends a message containing its storage and processing capacity and which streets are within its communication area to the other FOG (line 1 of Algorithm 2). A given FOG service or resource allocation to service a FOG service (lines 2–4). Propagation also occurs with the connection of a new vehicle and by changing a vehicle from one FOG to another (lines 5–7). This change is the one managed by the FOG to which the vehicle is currently connected. Thus, FOG checks for what is the next FOG that the vehicle will connect to. Moreover, allowing a unique knowledge of the number of resources each FOG has allows a better set of resources to serve a given service.
**Algorithm 2:**Communication Among FOGs1: send(Capacity,Streets)2: **if** (Servicefinalized) **then**3: send(Update_Capacity)4: **end if**5: **if** (Vehicleleaveorenter) **then**6: send(Update_Capacity)**7: end if**

### 3.2. Resource Allocation and Management

Since the resources are being made available by the vehicles and managed by the set of FOGs, once a vehicle cloud infrastructure was developed, it aims to offer services through the resources made available by the vehicles. Therefore, FORESAM needs to approach the problem of the allocation of resources to serve the requested services better. We can consider that there are vs. moving vehicles in the streets in an urban scenario and has several idle $r_{v}$ resources to understand the problem. These vehicles are connected to a set of FOGs *F* in which it has many idle resources *R*, which is composed of the sum of resources of clan FOG *f*. Therefore, each FOG *f* will have a set of $V_{f}$ vehicles connected to it, which share a total of $r_{f}$ resources.

A vs. vehicle needs to process information at a given moment, but its computational resources do not support processing such a task. To do so, it sends a request message to the formed infrastructure to find a set of resources available through the set of FOGs that could lend it their resources to perform its task. Therefore, the great challenge of FORESAM is to allocate the most considerable number of resources, meeting the demands of each service efficiently. For this, FORESAM uses multi-criteria mathematical methods, more precisely the elitist selection strategy [41], which takes a short time to decide due to the simplicity of decision calculations, consequently lowering the processing and energy consensus.

FORESAM aims to balance the load among services between the various FOGs, making the resources in vehicular clouds available while getting together the requirements of the resource of every service requested.

The proposed allocation mechanisms are implemented within the FOGs, which will select the best set of resources that meets the requested service. It will select the FOG, which can meet the minimum requirements of the requested service. For this, FORESAM uses the communication between the FOGs and vehicles to build its pool of resources. Furthermore, the communication between FOGs allows the exchange of information and resources between them.

The set of FOGs monitors the flow of information between the vehicles and the RSUs and updates the resource allocation and aggregation information available between all the FOGs in the set so that they all have the same information. The admission and departure of resources in the FOG are established by the connection between the vehicle and the FOG. Therefore, it is crucial to consider the time of execution of the service allocated and the service demand of each FOG to determine how processing and storage resources will be required to meet all demands.

As a result, the proposed mechanism uses as a decision parameter the memory used for the service, called storage; the total occupation time of processing to perform the service, called processing, and finally, the total execution time of the requested service, that is, the total time of use of the allocated resources, called service time. To exemplify, we can suppose that a vehicle requests a monitoring and route switching service, in which such service will be performed throughout its route, which will require a time of 30 min. Therefore, the service time to provide this service will be 30 min, assuming that this service needs approximately 300 MB of memory to store traffic information and processing time slots of approximately 5 min for the route change calculation.

Similar to these parameters, the decision mechanism implemented at FOGs can decide in which pool of resources only the requested service will be allocated. For this, all FOGs create a decision matrix to which FOG the service will be allocated. This matrix contains the storage, processing, and service time of each FOG. As FOGs exchange resource update information, the allocation of this matrix is the same for all FOGs.

A mechanism is used to fill in the matrix parameters that computes a priority value that a given parameter has over the other through the Analytic Hierarchy Process (AHP) [42] method. To obtain the AHP values, we consider that the service time has a higher priority over the other parameters. This is because such services will be performed over a vehicular network in which it has high mobility. Regarding the memory and the processor, we consider that the memory has a higher priority over processing because the vehicle has a smaller memory capacity than processing on its onboard computer. The decision mechanism builds a decision matrix in which the columns and lines indicate the metrics considered for decision making, i.e., service time, processing, and storing, to represent all pairwise comparisons, as shown in Table 2.

With the parameter that the FOG is monitoring, for example, the total processing time of its services, allocated with the multiplication of the processing priority weight calculated by AHP will result in the value assigned in the decision matrix. Therefore, the values obtained by AHP indicate which parameters have higher priority over others and serves as weights for each parameter in the decision matrix, as we can see in Equation (1).

(1) $$\begin{array}{c} If_{i}=fat_{i}\cdot st_{i} \end{array}$$

The influence factor $If_{i}$ (Equation (1)) is obtained by the multiplication of $fat_{i}$. This value is taken from Table 2, with the value of the computational resources that FOG is monitoring $st_{i}$.

Services performed by a vehicular cloud may have different requirements and priorities in their execution. For example, a service aimed at the safety of drivers must have a higher priority in allocation and execution than other services aimed at yellow pages or entertainment, as shown in Equation (2). Thus, we assign new weights for each type of service, safety, comfort, and entertainment, in this work. Where security service has weight 1 ($Serv_{1}$), comfort service weight 2 ($Serv_{2}$), and entertainment service has weight 3 ($Serv_{3}$). The weights of each service are multiplied with $If_{i}$, which results in the values placed in the decision matrix (Equation (4)). These values will be used to select the more appropriate FOG in the set of *n* FOGs.

(2) $$\begin{array}{c} Serv=\{Serv_{1},Serv_{2},Serv_{3}\}\; \end{array}$$

(3) $$\begin{array}{c} where\;Serv_{1}=1,\;Serv_{2}=2,\;Serv_{3}=3 \end{array}$$

(4) $$\begin{array}{c} M=\left[\begin{array}{ccc} Serv_{1}\cdot Inf_{i} & Serv_{2}\cdot Inf_{i} & Serv_{3}\cdot Inf_{i}\\ \vdots & \vdots & \vdots \\ Serv_{1}\cdot Inf_{n} & Serv_{2}\cdot Inf_{n} & Serv_{3}\cdot Inf_{n} \end{array}\right] \end{array}$$

As the values of the decision matrix are very irregular because they are different parameters, it is necessary to normalize these values so that a more precise decision can be made as to which FOG will be allocated to the service. Therefore, for standardization, we define the following:

(5) $$\begin{array}{c} M_{i,j}=\left(d_{i,j}-\bar{d_{J}}\right)/n\; \end{array}$$

where $d_{i,j}$ is the matrix value, and $\bar{d_{J}}$ is the arithmetic average contained in column *j* of the matrix.

Once the matrix parameters are normalized, we need to decide which will be the best FOG to allocate the service. To this end, we used the Euclidean distance between the attributes of the FOG node chosen *d* to the current conditions of the other FOG nodes *f*, based on Equation (6).

(6) $$\begin{array}{c} e=\sqrt{\sum_{j=1}^{n}{\left(d_{ij}-f_{ij}\right)}^{2}} \end{array}$$

Based on the higher value of *e*, the decision mechanism acknowledges the potential of a FOG node to allocate the resource to the service, meeting parameters requirements and considering the cost.

## 4. Performance Analysis

This section describes the validation of FORESAM and discusses the results obtained. For this, we divided the evaluation into two steps: (i) the Evaluation of the Allocation Mechanism of Resource Allocation in the Vehicular Cloud; which aims to assess the performance of the proposed allocation mechanism; (ii) the Evaluation of Communication among the Elements of FORESAM; aiming to evaluate the entire FORESAM communication and resource aggregation protocol.

FORESAM was implemented in the network simulator (NS3) [43], which all network traffic is carried out, that is, it carries out the communications of the FORESAM elements. Furthermore, we used the Simulator for Urban MOBIL (SUMO) [44]. It allows extracting vehicle mobility from a real trace.

**Figure 4:**
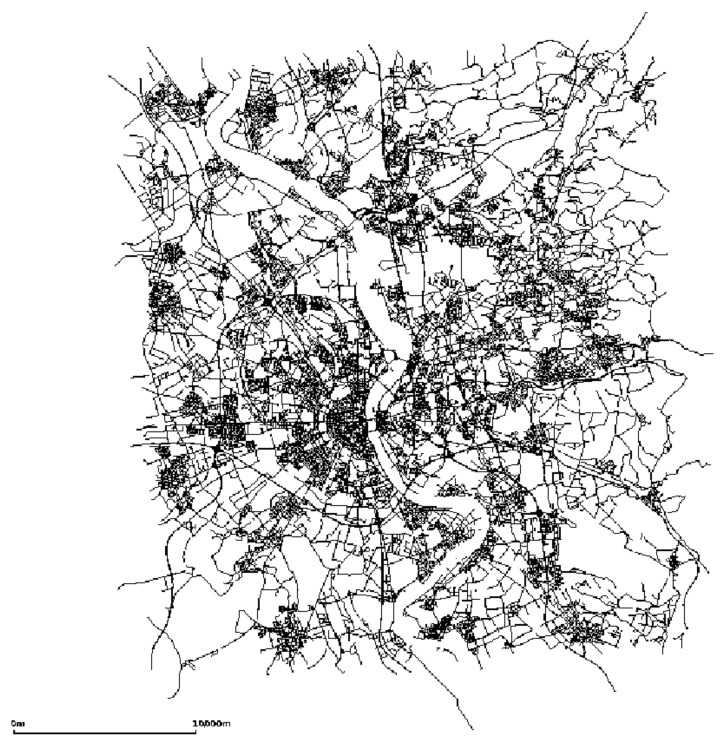
Cologne map.

The mobility of vehicles used the map of the city of Cologne, Germany (Figure 4). In this trace, we chose to use vehicle mobility between 6:00 a.m. and 8:00 a.m. on a weekday, covering an area of 400 km${}^{2}$ of the city. Thus, 120,000 vehicles were obtained. We chose this trace because it has both streets, avenues, and highways, which is very similar to a real environment.

In the simulation environment, we consider that FOG refers to an HP ProLiant ML110 G5 machine, in which it has the following configurations: Intel Xeon 3075, dual-Processor clocked at 2660 MHz, and 4 GB of RAM. Moreover, FOG used the 5G network to carry out its communication; the 5G used is the availability standard by NS-3. These settings allowed the entire map to be covered by 6400 FOGs. Due to the vehicle’s proximity to the FOG, the sending and response of requests tend to be fast. We assume that the cloud will be providing video streaming services, amber page services, and monitoring traffic.

Having several idle resources as a basis, the set of FOGs can provide a couple of services. In this paper, we consider that the FOG provides three services. A service will only be executed if it can count on a specific amount of allocated services. For example, we consider the offer of storage and processing by a cloud; services that require the allocation of between two and five resources.

Each vehicle has zero or two idle resources to share. Furthermore, we consider processing and storage as available resources. Therefore, the vehicle can be available for two, one, or zero resources. Moreover, the selection is made randomly, thus diversifying the number of resources in each FOG. For communication between vehicles, we consider a bit rate of 18 Mbit/s in the MAC layer and 2.2 mW as transmission power, allowing a coverage area of approximately 300 m when employing a two-ray ground propagation model [45]. For communication that comes with the FOGs, it uses the standard 5G. Table 3 shows the configuration and parameters that were used to perform the simulations.

The simulation scenarios were performed 35 times each, which made it possible to compute a 95% confidence interval.

### 4.1. Evaluation of Allocation Mechanism of Resource Allocation in the Vehicular Cloud

This section describes the assessment focusing on the allocation mechanism of resource allocation in the vehicular cloud. For this evaluation, we used four other mechanisms: (i) Best—ends up choosing the FOG where the resource allocation delivers the smallest number of idle resources; (ii) Worst—elects the FOG that resource allocation delivers the idlest resources; (iii) Random—chooses a FOG randomly among the FOG set; finally, (iv) Greedy—chooses the first with all the resources that the services have required.

Furthermore, we have used three metrics: (i) Services Attempt—signifies the service attempt amount in FOGs; (ii) Services blocked—represents the amount of services that was blocked until finding a FOG or was Denied; and (iii) Services Denied—represents the number of occurrences of non-allocation of services across the set of FOGs due to unavailability of resources.

**Figure 5:**
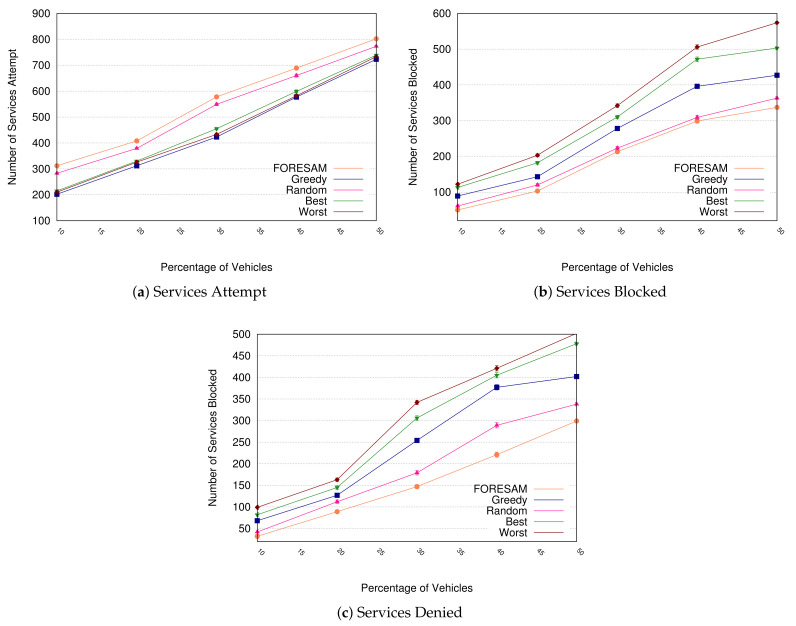
Performance analysis.

Figure 5a illustrates the number of times that a FOG has provided a service. As can be seen, when the comparison with other solutions was made, FORESAM achieved a considerable increase in the number of services attempted. When we perform a FORESAM comparison with the Best, Worst, and Greedy methods, we can see the number of services provided increased by approximately 49% when considering 50% of vehicles. In comparison to the Random method, we can realize the growth of 7%. This happens because the random algorithm selects, with no majority, the FOGs with the required number of terminations for the service every time.

Figure 5b illustrates the number of times a service starts to wait until the policy finds a FOG that meets the resources required by the service. Therefore, we can observe FORESAM obtaining a reduction of approximately 50% when compared to Greedy, Worst, and Best and decreasing about 8% compared to Random, and this happens due to the FORESAM placing among the first options the FOGs that can meet the number of resources requested by the service.

It can be seen in Figure 5c a certain number of required services being denied due to a low number of resources in the set of FOGs that attempt the service requested. FORESAM reduced approximately 42% in comparison with Best, Greedy, and Worst and also a reduction of 5% in comparison with Random, and this is possible because FORESAM allows a better balance of the load of resources within the FOG, i.e., Best, Worst and Greedy permits a disorderly allocation of resources.

Summarizing, FORESAM allows attempting a tremendous amount of service, provides a decrease in the amount of services blocked, as well as a reduced number of discarded services due to its decision mechanism in the allocation of resources.

### 4.2. Evaluation of Communication among Elements of FORESAM

In this section, we describe the assessment focusing on the impact that communication and resource aggregation and allocation could have on the network.

We used three metrics for this evaluation: (i) the amount of control messages; (ii) the delay in delivering messages; and (iii) the network packet loss percentage. As a comparison, we used the work of Refaat et al. [33] and the communication protocol, A-STAR [46].

**Figure 6:**
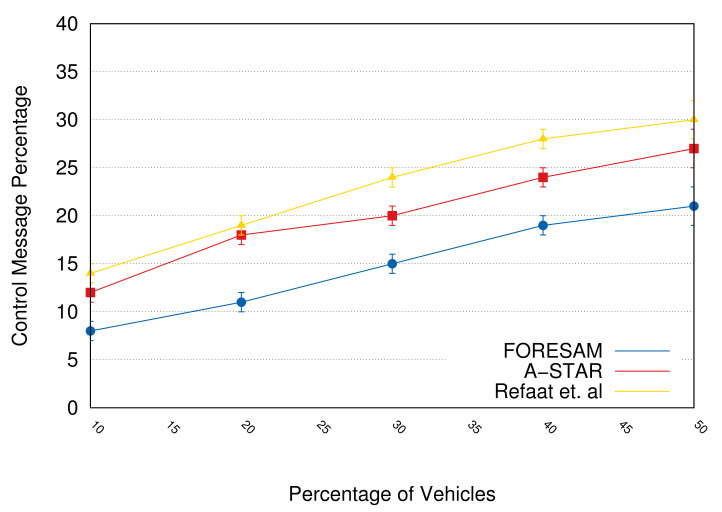
The percentage of control messages.

Figure 6 presents the network control message percentage. FORESAM had a smaller overhead because the proposed protocol reduced the message number to perform the aggregation of resources, and the connection with the vehicle is active. FORESAM achieved an approximated reduction of 15% compared to the Refaat et al.’s protocol and approximately 10% compared to A-STAR when we observe a growth in the amount of vehicles (50% of vehicles participating). However, a smaller reduction happens with a smaller amount of vehicles participating (10% of the vehicles participating) because the number of resources to serve the services is smaller, reducing the number of control messages as a consequence. This reduction had a significant impact when we analyzed the packet loss percentage and the average delay in messages delivering.

**Figure 7:**
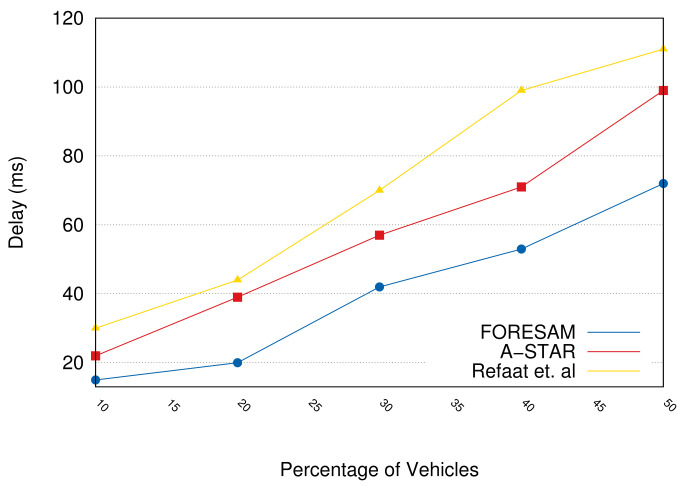
The average delay in delivering messages.

Figure 7 presents the average delays in the messages delivered. We can see that FORESAM achieved an approximately 20% reduction. This happens because the allocation decision policy in which most resources were managed by the same FOG started the connection. With a greater amount of vehicles (50% of vehicles), FORESAM can reduce approximately 30% compared to Refaat et al. protocol and 25% compared to the A-STAR.

**Figure 8:**
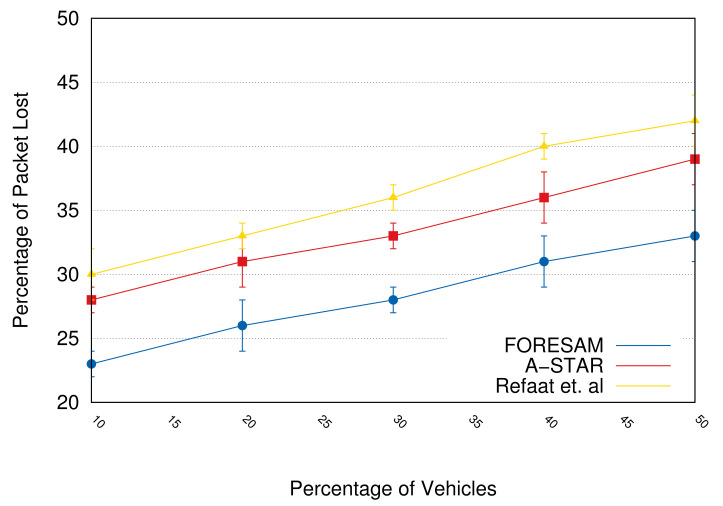
The percentage of packet loss.

Figure 8 shows the packet loss percentage on the network. FORESAM has a lesser amount of packet loss as there is less packet flow over the network. When analyzed at the point with the largest number of participating vehicles (50% vehicles), we can realize a 13% reduction. However, the reduction is only 10% if analyzed with a fewer number of vehicles.

To summarize, the architecture proved to be efficient because FORESAM reduced the number of control messages by 10% on the network, the average time of delivering information reduced by 20%, as well as the loss of information reduced by approximately 10%.

## 5. Conclusion and Future Work

During this research, the importance of ITS for the improvement of traffic management systems is investigated. The goal is to improve the quality of transport and make the trip more comfortable and safer for drivers and passengers. In this context, it is worth noting that mobile clouds can assist the ITS in handling the computer resource management problem. However, resource allocation management in an ITS is not trivial due to vehicular network characteristics, such as high mobility and dynamic topology.

We proposed FORESAM, which addresses the vehicular cloud problem with resource allocation. For this, we consider that clouds are composed of a FOG set with vehicles that have resources. Such resources can be shared, thus allowing the growth of the number of services presented by the cloud. FORESAM was modeled with mathematical methods to enable optimal decision making related to resource allocation in order to maximize resource utilization in the cloud. In other words, a mechanism for resources management and allocation was modeled based on a set of FOGs that control the vehicle’s cloud resources in the urban environment. The simulation results indicate that the FORESAM enables a higher quantity of services, offering a reduced number of service locks due to its precision and a reduced quantity of discarded services due to its mechanism of allocating resources. Furthermore, its resource allocation is more balanced and provided a smaller amount of discarded services.

In future work, we will improve the proposed mechanism by considering other parameters and scenarios and investigating its effects on resource allocation. Still, we intend to propose new resource allocation mechanisms in a FOG computing environment in the context of ITS.

## Figures and Tables

**Table 1:** Features comparison with related work.

Works	Res. Allocation		Service	Mode	Elements	Allocation Method
	Roadside	Vehicle	Dif. Service			
Garg et al. [39]		X		Decentralized	Vehicles	Optimization
Meneguette et al. [23]		X		Decentralized	Vehicles	SMDP
Hattab et al. [37]		X		Centralized	Vehicles	Querry
Da Costa et al. [36]		X		Centralized	Vehicles	Heuristic
Tao et al. [24]	X			Centralized	RSU	Optimization
Yu et al. [21]	X			Centralized	RSU	Optimization
Tang et al. [38]	X			Centralized	RSU	Heuristic
**FORESAM**	X	X	X	Hibridy	FOGs and Vehicles	AHP

**Table 2:** Influence Factor.

Factor	Service Time	Storage	Processing
Service Time	1	2	3
Storage	1/2	1	3
Processing	1/3	1/3	1

**Table 3:** Simulation parameters.

Parameter	Value
Communication RSU	5G
Transmission power	2.2 mW
Transmission range	300 m
Bit rate	18 Mbit/s
Beacons time	0. 5 s
Runs	35
Confidence Interval	95%
